# The Bournville Productions

**Published:** 1920-12-11

**Authors:** 


### The Bournville Productions.

We have had the pleasure of testing a series of Cadbury's latest productions from Bournville, and find that, apart from living up to the already world-famous reputation of this company's manufactures, the samples submitted to us appear both in variety and purity to deserve strong recommendation for the sick-room and the home.

